# Comparison of Myelodysplastic Syndrome Prognostic Scoring Systems

**DOI:** 10.4274/tjh.2014.0455

**Published:** 2016-05-16

**Authors:** Özlen Bektaş, Ayşegül Üner, Eylem Eliaçık, Burak Uz, Ayşe Işık, Sezgin Etgül, Süreyya Bozkurt, İbrahim Celalettin Haznedaroğlu, Hakan Göker, Nilgün Sayınalp, Salih Aksu, Haluk Demiroğlu, Osman İlhami Özcebe, Yahya Büyükaşık

**Affiliations:** 1 Hacettepe University Faculty of Medicine, Department of Hematology, Ankara, Turkey; 2 Hacettepe University Faculty of Medicine, Department of Pathology, Ankara, Turkey

**Keywords:** myelodysplastic syndrome, International Prognostic Scoring System, MD Anderson Prognostic Scoring System, World Health Organization Classification-Based Prognostic Scoring System, Revised International Prognostic Scoring System

## Abstract

**Objective:**

Myelodysplastic syndrome (MDS) is a clonal hematopoietic stem cell disease. Patients are at risk of developing cytopenias or progression to acute myeloid leukemia. Different classifications and prognostic scoring systems have been developed. The aim of this study was to compare the different prognostic scoring systems.

**Materials and Methods:**

One hundred and one patients who were diagnosed with primary MDS in 2003-2011 in a tertiary care university hospital’s hematology department were included in the study.

**Results:**

As the International Prognostic Scoring System (IPSS), World Health Organization Classification-Based Prognostic Scoring System (WPSS), MD Anderson Prognostic Scoring System (MPSS), and revised IPSS (IPSS-R) risk categories increased, leukemia-free survival and overall survival decreased (p<0.001). When the IPSS, WPSS, MPSS, and IPSS-R prognostic systems were compared by Cox regression analysis, the WPSS was the best in predicting leukemia-free survival (p<0.001), and the WPSS (p<0.001) and IPSS-R (p=0.037) were better in predicting overall survival.

**Conclusion:**

All 4 prognostic systems were successful in predicting overall survival and leukemia-free survival (p<0.001). The WPSS was found to be the best predictor for leukemia-free survival, while the WPSS and IPSS-R were found to be the best predictors for overall survival.

## Abstract (tr)

**Amaç:**

Miyelodisplastik sendrom (MDS) klonal bir hematopoetik kök hücre hastalığıdır. Hastalarda sitopeni veya akut miyeloid lösemi gelişmesi riski söz konusudur. Farklı sınıflandırma ve prognostik skorlama sistemleri geliştirilmiştir. Bu çalışmanın amacı, farklı prognostik skorlama sistemlerinin karşılaştırılmasıdır.

**Gereç ve Yöntemler:**

Üçüncü basamak üniversite hastanesi, hematoloji bölümünde 2003-2011 yılları arasında tanı alan 101 primer MDS hastası çalışmaya dahil edildi.

**Bulgular:**

Uluslararası Prognostik Skorlama Sistemi (UPSS), Dünya Sağlık Örgütü Sınıflandırması Bazlı Prognostik Skorlama Sistemi (DPSS), MD Anderson Prognostik Skorlama Sistemi (MPSS) ve yeniden düzenlenmiş UPSS (UPSS-D) risk kategorileri arttıkça lösemisiz sağkalım ve toplam sağkalım azalıyordu (p<0,001). UPSS, DPSS, MPSS ve UPSS-R Cox regresyon analizi ile karşılaştırıldığında, DPSS’nin lösemisiz sağkalımı (p<0,001), DPSS (p<0,001) ve UPSS-D’nin (p=0.037) toplam sağkalımı daha iyi öngördüğü tespit edildi.

**Sonuç:**

Dört prognostik skorlama sistemi de toplam sağkalımı ve lösemisiz sağkalımı başarılı şekilde öngörüyordu (p<0.001). DPSS’nin lösemisiz sağkalımın, DPSS ve UPSS-D’nin toplam sağkalımın en iyi öngöreni olduğu tespit edildi.

## INTRODUCTION

Myelodysplastic syndromes (MDSs) are a heterogeneous group of clonal hematopoietic stem cell disorders with heterogeneous morphological, clinical, and survival characteristics. Common features include cytopenia(s), dysplasia of one or more major myeloid series, ineffective hematopoiesis, and an increased risk of acute myeloid leukemia [1].

In 1982, the first classification of MDS was developed by the French-American-British (FAB) group. This was a morphological classification based on the degree of dysplasia and blasts in the bone marrow, without biological basis [2]. The World Health Organization (WHO) rearranged the classification of MDS-FAB in 2001 and 2008. Several parameters with prognostic significance were added in the 2008 version: number of cytopenias, dysplasia in one or more series, and presence of genetic abnormalities [3].

Following diagnosis and classification of MDS, prognostic staging should be made to plan the treatment [4]. The International MDS Risk Analysis Workshop developed the International Prognostic Scoring System (IPSS), recognizing the bone marrow blast percentage, cytogenetic status, and number and degree of cytopenias as the most important prognostic markers in MDS [5]. The IPSS is the most widely used prognostic scoring system [4]. It was designed based on untreated and primary MDS patients [5]. The WHO category, cytogenetics, and transfusion requirements were identified as the most important prognostic indicators in MDS and the WHO Classification-Based Prognostic Scoring System (WPSS) was developed by Malcovati et al. [6]. The WPSS was also designed based on untreated patients and it does not include secondary MDS patients. Kantarjian et al. developed a new classification model to overcome the limitations existing in both prior prognostic systems of MDS, which includes patients’ performance status, age, number and degree of cytopenias, cytogenetics, bone marrow blast percentage, and transfusion needs [7]. The MD Anderson Prognostic Scoring System (MPSS) is a system that can be applied to primary and secondary MDS and chronic myelomonocytic leukemia (CMML). The International Working Group for Prognosis in MDS project was initiated due to limitations of the IPSS and the revised IPSS (IPSS-R) was developed. The IPSS-R considers bone marrow blast percentage, cytogenetics, and number and degree of cytopenias. This prognostic system also does not include secondary MDS and was developed based on untreated patients [8].

In this study, we aimed to compare the different prognostication systems and determine the most appropriate system for routine clinical practice.

## MATERIALS AND METHODS

One hundred and one patients who were diagnosed with primary MDS during 2003-2011 and suitable for all of the prognostication systems were included in the study. We used 101 routinely managed patients regardless of whether they were on MDS-specific treatment or not. Patient information was accessed from patient chart reviews. Each patient was categorized according to the MDS-FAB and 2001 WHO classification systems according to their bone marrow aspiration and biopsy specimens. We did not use 2008 WHO classification since the WPSS was validated only for the 2001 WHO classification system. Criteria for inclusion were: age >18 years, primary MDS patients, and marrow and peripheral blood blast counts of <20%. Exclusion criteria were: CMML, secondary MDS, and marrow or peripheral blood blast counts of ≥20%.

To analyze the prognosis, we used 4 different prognostic systems: the IPSS, WPSS, MPSS, and IPSS-R. Leukemia transformation and death were recorded as events and the first developed event was recorded. Event-free survival was defined as the duration from the time of diagnosis until the time of developing an event or the last follow-up time, leukemia-free survival (LFS) was defined as the duration from the time of diagnosis until the time of developing leukemia (marrow or peripheral blood blast count of ≥20%) or the last follow-up time, and overall survival (OS) was defined as the duration from the time of diagnosis until death or the last follow-up time. Last follow-up date and condition were recorded as the last condition.

This investigation was approved by the Local Ethics Committee of Hacettepe University.

### Statistical Methods

Data analysis was performed using SPSS 11.5 for Windows. Continuous data were presented as mean ± standard deviation or median (range). Categorical data were presented as numbers and percentages. For the IPSS, WPSS, MPSS, and IPSS-R, the LFS, OS, and life expectancy were evaluated with Kaplan-Meier survival analysis using the log-rank test. Life expectancy; 1-, 3-, and 5-year survival rates; and 95% confidence intervals (CIs) were calculated for each variable category. The prediction capacities of the IPSS, WPSS, MPSS, and IPSS-R for LFS and OS were compared with multivariate Cox proportional hazard regression analysis. For each variable, the hazard ratios and 95% CIs were calculated.

A value of p<0.05 was considered statistically significant.

## RESULTS

### Patient Characteristics

The present study consisted of 101 patients; 44 of them (43.6%) were male and 57 (56.4%) were female. The mean age of the patients was 64±14.77 years. Transfusion support was given to 26 (23%) patients; hypomethylating agents were used in 21.2% of patients (n=24; 23 of them were on 5-azacytidine and 1 was on decitabine), lenalidomide in 0.9% of patients (n=1), and erythropoietin in 2.7% of patients (n=3); and 4.4% (n=5) of patients had undergone allogeneic bone marrow transplantation. The follow-up period for the patients ranged between 0 and 92 months with an average of 21.2 months. Cytogenetic classification of the patients according to the IPSS was as follows: 66 (58.4%) of good risk, 17 (15%) of intermediate risk, and 18 (15.9%) of poor risk. MDS subgroup distributions according to both the MDS-FAB classification and the 2001 WHO classification are shown in Table 1.

Patients were evaluated by 4 different prognostic systems. Accordingly, the risk distributions of the patients are shown in Table 2.

During the follow-up period, 34.7% (n=35) of patients experienced an event. The first event was leukemic transformation in 20.8% of the patients (n=21), while death was the first event in 13.9% (n=14) of the patients. Median time to event was 15.25 months. Median leukemic progression time was 8.28 months. Total death rate was 29.7% (n=30). The estimated OS and LFS durations were 55.93±10.19 and 56.52±10.29 months, respectively.

**Figure 1:**
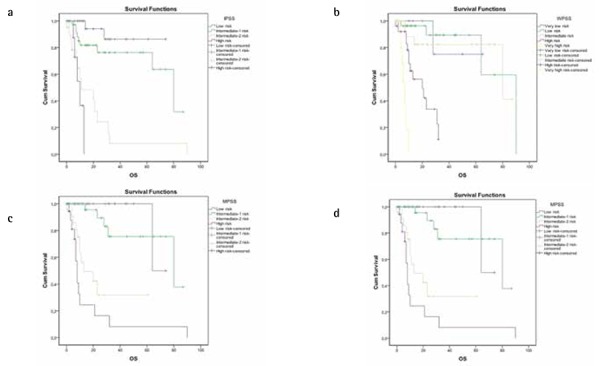
Kaplan-Meier curves show rates of overall survival (OS) for International Prognostic Scoring System (IPSS) (a), World Health Organization-Based Prognostic Scoring System (WPSS) (b), MD Anderson Prognostic Scoring System (MPSS) (c), and Revised International Prognostic Scoring System (IPSS-R) (d).

In all 101 patients the average life expectancy was 55.9 months (95% CI: 45.77-66.09), and 1-, 3-, and 5-year OS rates were found as 77.5%, 57.5%, and 57.5%, respectively. The OS and median survival times were significantly reduced as the degree of risk increased regardless of which classification system was used (p<0.001) (Table 3, Figures 1a, 1b, 1c, 1d).

**Figure 2:**
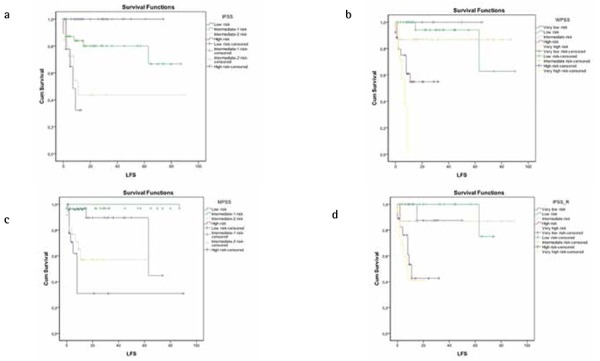
Kaplan-Meier curves show rates of leukemia-free survival (LFS) for International Prognostic Scoring System (IPSS) (a), World Health Organization Classification-Based Prognostic Scoring System (WPSS) (b), MD Anderson Prognostic Scoring System (MPSS) (c), and Revised International Prognostic Scoring System (IPSS-R) (d).

In all 4 classification systems, the LFS was reduced as the degree of risk increased (p<0.001). The 1-, 3-, and 5-year leukemia survival rates in all subjects were 76%, 60.1%, and 60.1%, respectively, and the average LFS time was found to be 56.52 months (95% CI: 46.2-66.8) (Table 4, Figures 2a, 2b, 2c, 2d).

When the efficacies of the IPSS, WPSS, MPSS, and IPSS-R prognostic systems in predicting LFS were compared, the WPSS showed the best performance (p<0.001, hazard ratio [HR]: 2.1, 95% CI: 1.543-2.858). The WPSS (p<0.001, HR: 2.461, 95% CI: 1.812-3.343) and IPSS-R (p=0.037, HR: 1.460, 95% CI: 1.024-2.081) systems were better than the others in predicting OS.

## DISCUSSION

The current prognostication systems have been criticized for some specific properties. They were developed in untreated cohorts and they have generally not been tested in treated cohorts except for the IPSS-R. Neukirchen et al. demonstrated the value of the IPSS-R for patients treated with induction chemotherapy and/or allogeneic stem cell transplantation in their validation study [9]. Currently there are many widely available treatment alternatives in MDS. Therefore, we thought that these systems should be tested in a modern routinely managed MDS cohort. The IPSS and IPSS-R are mostly criticized because they were developed in untreated patient cohorts that do not reflect current patient profiles [10]. The MPSS is mainly criticized for the inclusion of secondary and therapy-related MDS and MDS/myeloproliferative disease cases, which are now considered separate entities [11,12]. The WPSS was initially criticized for arbitrariness of transfusion dependence. However, it was revised to include stable hemoglobin thresholds instead of this arbitrary definition [13]. It is still criticized for low reproducibility of WHO classification of subentities with low blast counts.

In spite of these critiques, there is no doubt that these systems are useful in routine clinical practice. But which one(s) deserve the most credit?

In our study 101 MDS patients appropriate for all prognostic systems were evaluated with the IPSS, WPSS, MPSS, and IPSS-R. The median age of the patients was 64 years, which is lower than in Western populations; younger age at diagnosis was also seen in some Asian countries, as Matsuda et al. and Kuendgen et al. demonstrated [1,14]. This is the first study to compare these 4 prognostic scoring systems in MDS. All 4 prognostic systems were successful in predicting OS and LFS (p<0.001). When the systems were compared, the WPSS was found to be the best predictor for LFS, while the WPSS and IPSS-R were found to be the best predictors for OS. Equal efficacies of IPSS-R and WPSS in our practice implies that our hematopathologists are quite capable of separating single-lineage dysplasia from multilineage dysplasia and refractory anemia with excess blasts (RAEB)-I from RAEB-II. Unfortunately, this capability may not be available in every setting.

There are several studies that compared prognostic scoring systems. Voso et al. compared the IPSS, WPSS, and IPSS-R in their IPSS-R validation study and found that the IPSS-R predicted OS better than the other systems [15]. Reis-Alves et al. showed that only IPSS-R score was an independent risk factor in terms of OS in their comparison of the IPSS, WPSS, and IPSS-R [16].

In our study, the WPSS and IPSS-R may have estimated OS better since the hemoglobin cut-off was accepted as lower than in the other systems in both these scoring systems (<9 g/dL in males and <8 g/dL in females for WPSS; 8-10 g/dL [1 point] and <8 g/dL [1.5 point] for IPSS-R). This may be especially true for low-risk patients since the main predictor of mortality is marrow failure in low-risk patients and leukemic transformation in high-risk patients. When the advanced age and frailty of many MDS patients are taken into consideration, the lower hemoglobin threshold may better reflect the impact of anemia on health. In our study, the WPSS was found to be best in reflecting LFS. This may be due to the fact that it depends on the MDS-WHO classification. This classification reflects leukemia transformation risk very well [17,18].

The MPSS is a dynamic scoring system like the WPSS and predicts survival at any time during follow-up. It can be used for chronic myelomonocytic leukemia and secondary MDS if prognostic assessment is required [7].

This study has some handicaps inherent to its retrospective nature. Additionally, it would be better to include a higher number of patients in future analyses.

## Ethics

Ethics Committee Approval: This investigation was approved by the Local Ethics Committee of Hacettepe University, Informed Consent: Not applicable (retrospective study).

## Figures and Tables

**Table 1:**
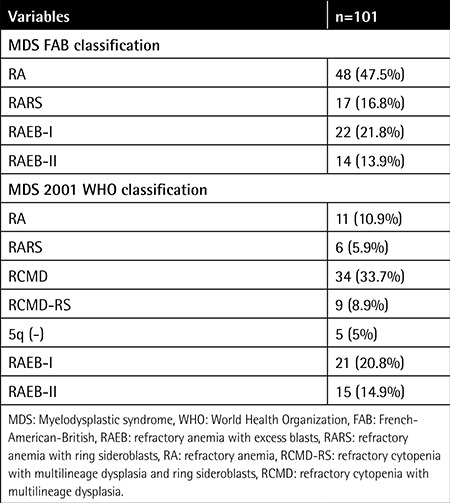
Distribution of patients according to French-American-British and World Health Organization 2001 classification systems.

**Table 2:**
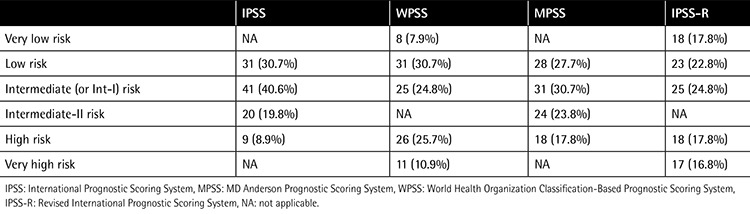
Distribution of patients by risk groups.

**Table 3:**
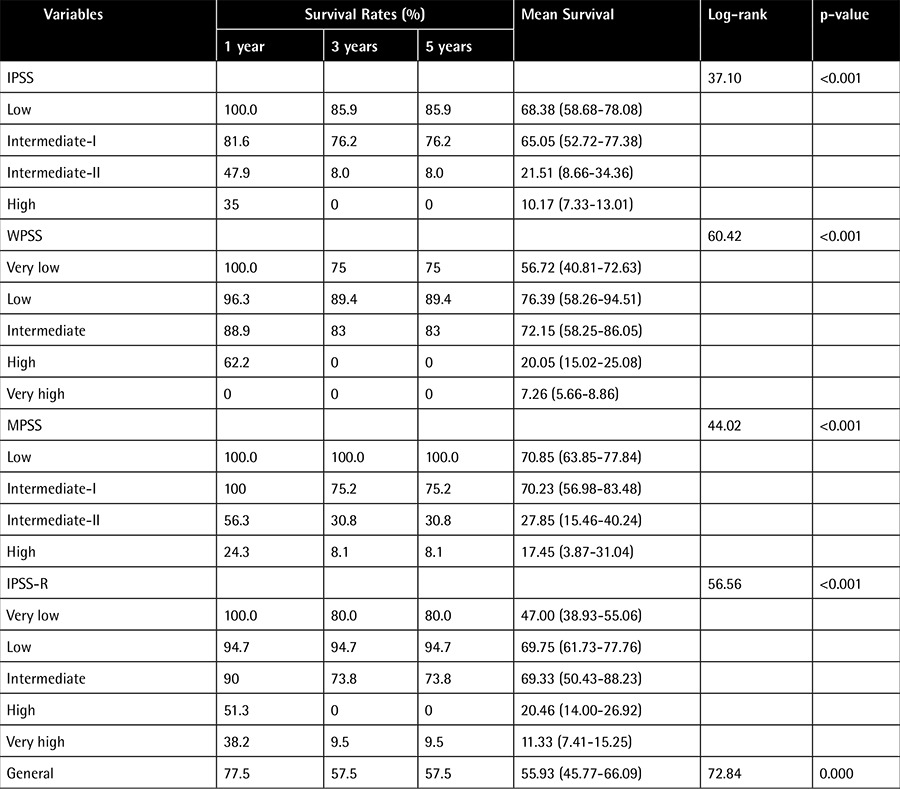
Overall survival according to International Prognostic Scoring System, World Health Organization Classification-Based Prognostic Scoring System, MD Anderson Prognostic Scoring System, and Revised International Prognostic Scoring System.

**Table 4:**
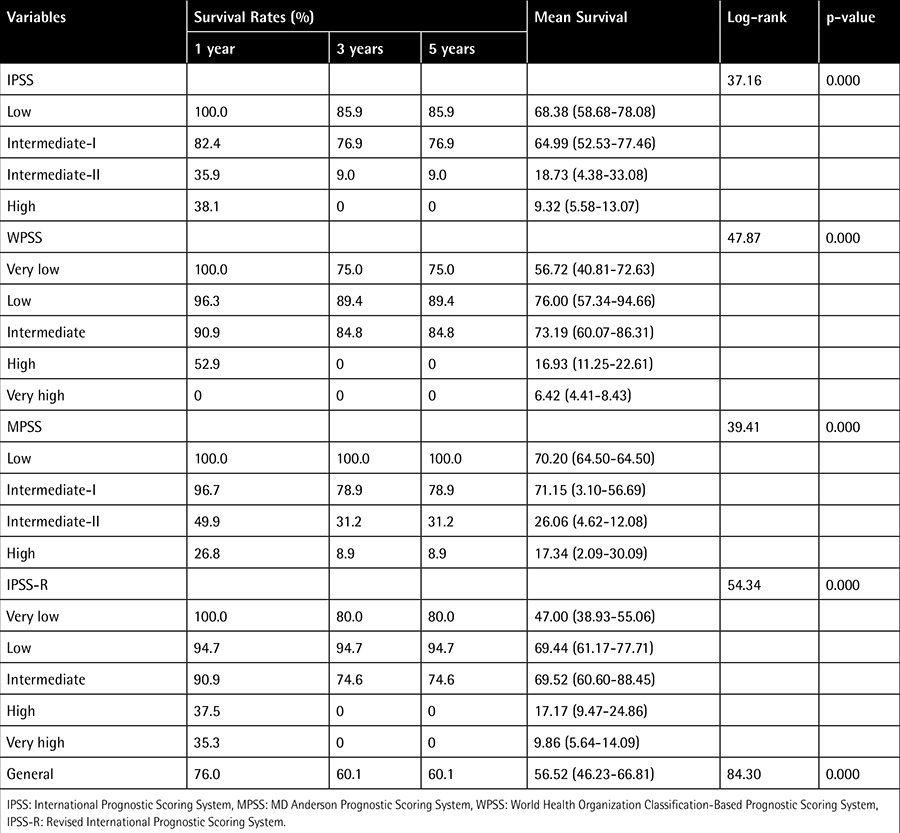
Leukemia-free survival according to International Prognostic Scoring System, World Health Organization Classification-Based Prognostic Scoring System, MD Anderson Prognostic Scoring System, and Revised International Prognostic Scoring System.
